# An *Abies* Extract Containing Nonvolatile Polyphenols Shows Virucidal Activity against SARS-CoV-2 That Is Enhanced in Increased pH Conditions

**DOI:** 10.3390/pathogens12091093

**Published:** 2023-08-28

**Authors:** Imane Maaroufi, Dulamjav Jamsransuren, Koh Hashida, Sachiko Matsuda, Haruko Ogawa, Yohei Takeda

**Affiliations:** 1 Graduate School of Animal and Veterinary Sciences and Agriculture, Obihiro University of Agriculture and Veterinary Medicine, Obihiro 080-8555, Japan; imanemaaroufivet@gmail.com; 2 Research Center for Global Agromedicine, Obihiro University of Agriculture and Veterinary Medicine, Obihiro 080-8555, Japan; jduuya@obihiro.ac.jp (D.J.); chaka@obihiro.ac.jp (S.M.); 3 Department of Forest Resources Chemistry, Forestry and Forest Products Research Institute, Tsukuba 305-8687, Japan; koh@ffpri.affrc.go.jp; 4 Department of Veterinary Medicine, Obihiro University of Agriculture and Veterinary Medicine, Obihiro 080-8555, Japan; hogawa@obihiro.ac.jp

**Keywords:** *Abies*, conifer, COVID-19, phytochemicals, polyphenols, SARS-CoV-2, virucidal

## Abstract

Researching the beneficial health properties of wood byproducts can prevent wastage by turning them into valuable resources. In this study, the virucidal activity of two extracts from *Abies sachalinensis* byproducts, ASE1, and ASE2, against severe acute respiratory syndrome coronavirus 2 (SARS-CoV-2) was investigated. ASE1 is rich in monoterpenoid volatile compounds, whereas ASE2 contains nonvolatile polyphenols. SARS-CoV-2 solutions were mixed with ASE1 or ASE2, and viral titer reduction was evaluated. At their original acidic pH, ASE2 showed stronger virucidal activity than ASE1. The virucidal activity of ASE2 was also significantly enhanced when pH was increased to neutral or basic, which was not the case for ASE1. At a neutral pH, ASE2 induced statistically significant viral titer reduction in 1 min. HCl and NaOH solutions, which had a pH close to that of acidic and basic ASE2 test mixtures, respectively, exhibited no virucidal activity against SARS-CoV-2. Among the SARS-CoV-2 variants, Omicron showed the highest vulnerability to ASE2. Western blotting, RT-PCR, and electron microscopic analysis revealed that neutral ASE2 interacts with SARS-CoV-2 spike proteins and moderately disrupts the SARS-CoV-2 genome and viral envelope. These findings reveal the virucidal potential of ASE2.

## 1. Introduction

Characterized by needle-shaped leaves and seed cones, conifers are gymnosperm trees distributed worldwide that have shown pharmacological importance in previous years. The three major phytochemical groups found in conifers are terpenoids, alkaloids, and polyphenols, which have antimicrobial, antitumoral, and antioxidant properties [1,2,3,4]. These compounds are found in varying proportions depending on the family, genus, or species of conifer. The *Abies* genus of the Pinaceae family includes 50 species distributed in Asia, Europe, North Africa, and Central and Northern America [5]. *A. sachalinensis*, an important species of oroboreal coniferous forests, is found in the insular oroboreal belt of temperate Asia, from Sakhalin island in the north to Hokkaido island in the south [6]. *A. sachalinensis* is the most common conifer species in Hokkaido, where it forms mixed forests with *Picea jezoensis*, another member of the Pinaceae family [6].

For decades, the wood industry in Hokkaido steadily declined because of population aging, subsequent labor force reduction, and wood price reductions. At present, wood industry byproducts, which are essentially barks, branches, and needles of *A. sachalinensis*, have little to no economic value. However, antifungal activity from a wood extract [7], antibacterial activity from a needle extract [8], and the antitumoral effects of spiro-biflavonoids isolated from the bark [9] of *A. sachalinensis* have been reported. Therefore, *A. sachalinensis* byproducts offer promising sources of antimicrobial and chemopreventive agents, and studying their beneficial health properties could turn them into valuable resources, reducing wastage.

In the previous decade, an aqueous extract of *A. sachalinensis* branches and leaves was found to possess virucidal activity against influenza A virus [10]. Similar to influenza A virus, severe acute respiratory syndrome coronavirus 2 (SARS-CoV-2) is an enveloped single-stranded RNA virus [11]. It is the causative agent of coronavirus disease 2019 (COVID-19), which has been the greatest public health concern over the past three years. Transmission models suggest that the currently circulating SARS-CoV-2 Omicron variant has a higher transmissibility than influenza A virus [12], and COVID-19 is expected to remain endemic as a result. Despite the decreased pathogenicity of the Omicron variant [13], the SARS-CoV-2 mortality rate remains high among vulnerable populations, such as the elderly and immunocompromised hospitalized patients [14,15].

SARS-CoV-2 spread is currently being controlled via the use of alcohol-based disinfectants; two hand-sanitizer formulations recommended by the WHO are currently being used around the world [16]. Alcohol-based disinfectants are inexpensive and can be used on both skin and inanimate surfaces. Significant SARS-CoV-2 inactivation was reported for WHO-recommended hand sanitizer formulations within a contact time of only 30 s [17]. However, the regular use of alcohol-based disinfectants is irritating to the skin, and can induce adverse skin reactions such as inflammation, skin dryness, and contact dermatitis [18]. Subsequently, alternatives such as plant-derived virucidal agents and soaps have attracted attention [19]. Unlike alcohol-derived disinfectants, many plant-derived virucidal agents are regarded non-toxic and biocompatible with human skin [19,20]. Plant extracts and essential oils have also found applications as safer alternatives to chemical disinfectants in the food industry [21]. Furthermore, virucidal agents derived from wood byproducts can be regarded eco-friendly, as they reduce waste.

Thus, there is an opportunity to combine the need to provide safer plant-derived virucidal agents for SARS-CoV-2 control among vulnerable populations with the need to valorize wood byproducts and reduce their wastage. In this study, the virucidal activity of two extracts from *A. sachalinensis* branches and leaves against SARS-CoV-2 was evaluated.

## 2. Materials and Methods

### 2.1. Viruses and Cells

Multiple SARS-CoV-2 strains: 2019-nCoV/Japan/TY/WK-521/2020 (ancestral strain, Pango lineage: A, GISAID ID: EPI_ISL_408667), hCoV-19/Japan/QHN001/2020 (Alpha strain, B.1.1.7, EPI_ISL_804007), hCoV-19/Japan/TY8-612-P1/2021 (Beta strain, B.1.351, EPI_ISL_1123289), hCoV-19/Japan/TY7-501/2021 (Gamma strain, P.1, EPI_ISL_877769), hCoV-19/Japan/TY11-927-P1/2021 (Delta strain, AY.122, EPI_ISL_2158617), and hCoV-19/Japan/TY38-873P0/2021 (Omicron strain, BA.1.18, EPI_ISL_7418017) were obtained from the National Institute of Infectious Diseases (Tokyo, Japan). Vero E6/TMPRSS2 cells were obtained from the Japanese Collection of Research Bioresources (No. JCRB1819, Osaka, Japan) and passaged in a Dulbecco’s modified Eagle’s minimal essential medium (DMEM) (Nissui Pharmaceutical Co., Ltd., Tokyo, Japan) supplemented with 10% fetal bovine serum, 2 mM L-glutamine (FUJIFILM Wako Pure Chemical Co., Osaka, Japan), 0.15% NaHCO_3_ (FUJIFILM Wako Pure Chemical Co.), 2 µg/mL amphotericin B (Bristol-Myers Squibb Co., New York, NY, USA), 100 µg/mL kanamycin (Meiji Seika Pharma Co., Ltd., Tokyo, Japan), and 100 µg/mL G418 disulfate (Nacalai Tesque Inc., Kyoto, Japan). After inoculation with SARS-CoV-2, the cells were cultured for three days in a viral growth medium composed of DMEM supplemented with 1% fetal bovine serum, 2 mM L-glutamine, 0.15% NaHCO_3_, 2 µg/mL amphotericin B, and 100 µg/mL kanamycin.

### 2.2. A. sachalinensis Extracts and pH Adjustment

**Figure 1:**
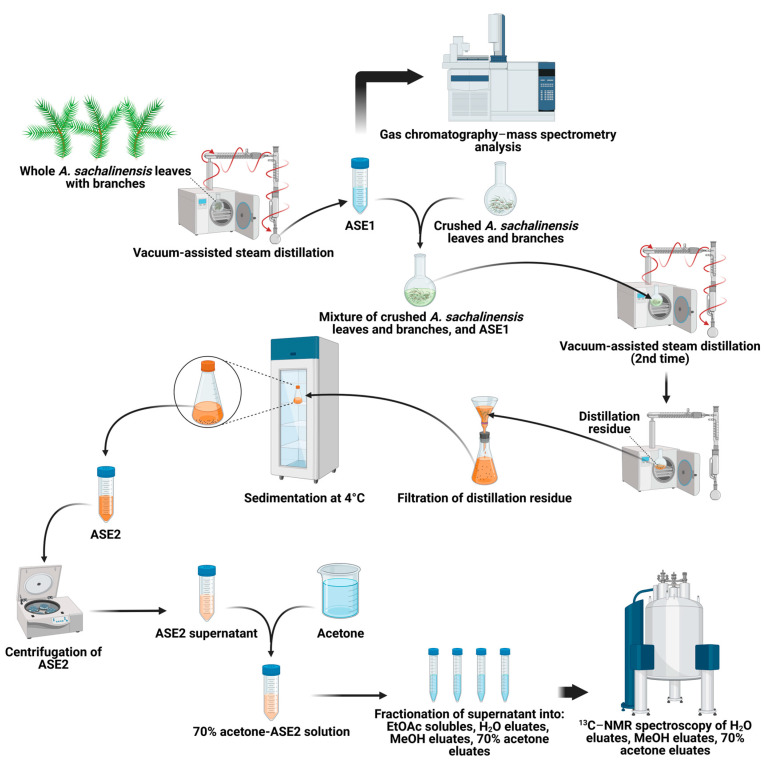
Major steps of the extraction process and analysis of ASE1 and ASE2.

Two *A. sachalinensis* extracts, ASE1, and ASE2, were obtained from Japan Aroma Laboratory Co., Ltd. (Tokyo, Japan). *A. sachalinensis* branches and leaves were obtained as byproducts of Hokkaido wood industry by Japan Aroma Laboratory Co., Ltd. A purified aqueous ASE1 extract was obtained through vacuum-assisted steam distillation of whole branches and leaves. Then, ASE1 was mixed with crushed *A. sachalinensis* branches and leaves, and vacuum-assisted steam distillation was performed again with the mixture. The distillation residue was collected, filtrated, and sedimented at 4 °C. The supernatant was collected and analyzed as ASE2. The composition of ASE1 was analyzed using gas chromatography–mass spectrometry. For the analysis of the composition of ASE2, this extract was centrifuged (10,000 rpm, 20 °C, 1 h) using a CR22GIII (Hitachi Koki Co., Tokyo, Japan) to remove H_2_O insolubles. Acetone was added to the supernatant solution to a final concentration of 70%, after which the solution was centrifuged (10,000 rpm, 20 °C, 1 h) to remove the 70% acetone insolubles. The soluble part of the 70% acetone solution was fractionated to ethyl acetate (EtOAc) solubles, H_2_O eluates, 50% methanol (MeOH) eluates, and 70% acetone eluates, in accordance with a method previously described by Liu et al. [22]. ^13^C–NMR spectra were recorded on an AVANCE 400 III HD spectrometer (Bruker Ltd., Billerica, MA, USA) at room temperature using acetone-d_6_/D_2_O (7/3, *v*/*v*) for 50% MeOH eluates and 70% acetone eluates, or using acetone-d_6_/D_2_O (1/9, *v*/*v*) for H_2_O eluates and the authentic sugars (sucrose, fructose, and glucose) (FUJIFILM Wako Pure Chemical Co.). The authentic sugars were used as references to interpret the ^13^C–NMR spectra. The major steps of the ASE1 and ASE2 extraction process and analysis are shown in Figure 1.

ASE1 and ASE2 are naturally acidic; ASE1 has a pH of 3.5, whereas ASE2 has a pH of 4.5. ASE1 is colorless, whereas ASE2 is of a brown–orange color. Although the direct virucidal impact of acidic pH is unclear, some solutions containing acidic compounds show virucidal properties against SARS-CoV-2 [23,24]. Therefore, to rule out the potential independent effects of acidity on SARS-CoV-2, pH 7.0 ASE1 (neutral ASE1), pH 7.0 ASE2 (neutral ASE2), and pH 9.0 ASE2 (basic ASE2) samples were prepared by adding 4N NaOH solution (FUJIFILM Wako Pure Chemical Co.).

### 2.3. Evaluation of the Virucidal Activity of ASE1 and ASE2 against SARS-CoV-2

SARS-CoV-2 solutions (viral titer: 7.0 log_10_ 50% tissue culture infective dose [TCID_50_]/mL) containing either ancestral or variant strains were mixed with nine volumes of acidic or neutral ASE1 or nine volumes of acidic, neutral, or basic ASE2. Negative controls were also prepared by mixing each SARS-CoV-2 solution with nine volumes of ultra-pure water (UPW). The estimated final pH of the mixture containing the virus solution and acidic ASE1 or ASE2 was pH 5.1, while that of the mixture containing basic ASE2 was 8.7. Since the actual pH of these mixtures could not be measured from the perspective of safe management of SARS-CoV-2, the pH was estimated by measuring the pH of mixtures of the same volume of virus-free solution (solvent of SARS-CoV-2 solution) mixed with acidic ASE1 or ASE2 or basic ASE2. Acidic and basic pH controls were also prepared by mixing the SARS-CoV-2 ancestral strain solution with nine volumes of HCl solution (pH 2.9) or NaOH solution (pH 10.0). The estimated final pH of the HCl control mixture (pH 5.1) was similar to that of acidic ASE1 or ASE2 mixtures, while the estimated final pH of the NaOH control mixture (pH 8.7) was similar to that of basic ASE2 mixture. These mixtures were incubated at 25 °C for from 1 min to 24 h and then inoculated to Vero E6/TMPRSS2 cells in a 10-fold serial dilution. After three days incubation at 37 °C, cytopathic effects were assessed, and the viral titer (log_10_ TCID_50_/mL) was calculated using the Behrens–Kärber method [25]. The detection limit was determined after assessing UPW, ASE1, and ASE2 for cytotoxicity on Vero E6/TMPRSS2 cells by CellTiter-Glo^®^ Luminescent Cell Viability Assay (Promega Co., Madison, WI, USA). ASE2 demonstrated mild cytotoxicity, and the detection limit of viral titer for virucidal activity evaluation was set to 2.25 log_10_ TCID_50_/mL. ASE1 and UPW did not show cytotoxicity, so the detection limit was set to 1.25 log_10_ TCID_50_/mL.

### 2.4. Western Blotting (WB)

To analyze the effect of ASE2 on spike (S) and nucleocapsid (N) proteins, the SARS-CoV-2 solution containing the ancestral strain was mixed with nine volumes of acidic or neutral ASE2 or UPW, and incubated at 25 °C for 24 h. Sodium dodecyl sulfate (SDS)-buffer with 2-mercaptoethanol (FUJIFILM Wako Pure Chemical Co.) was added to the mixtures, and this was heated to 100 °C for 2 min. Then, SDS-polyacrylamide gel electrophoresis was performed using 12% polyacrylamide gel. Precision Plus Protein™ All Blue Prestained Protein Standards (Bio-Rad Laboratories Inc., Hercules, CA, USA) were used as molecular mass markers. The proteins inside the acrylamide gel were transferred to a polyvinylidene difluoride (PVDF) membrane (Bio-Rad Laboratories Inc.) for WB. The membrane was probed with either SARS-CoV-2 (2019-nCoV) Spike Antibody, Rabbit PAb, Antigen Affinity Purified (Catalog No. 40591-T62, Sino Biological Inc., Beijing, China), or SARS-CoV-2 (2019-nCoV) Nucleocapsid Antibody, Rabbit MAb (Catalog No. 40143-R019, Sino Biological Inc.); with Anti-Rabbit IgG (γ-chain specific)–Peroxidase antibody, Mouse monoclonal (Catalog No. A1949, Clone: RG-96, Sigma-Aldrich Inc., Saint Louis, MO, USA) used as a secondary antibody. Finally, the membrane was developed using an ECL Prime WB Detection Reagent (GE Healthcare Ltd., Chicago, IL, USA), and chemiluminescence was detected using the LAS-3000 Imaging System (FUJIFILM Co., Ltd., Tokyo, Japan).

### 2.5. Reverse Transcription (RT)-PCR

Conventional RT-PCR and real-time RT-PCR were performed. The SARS-CoV-2 solution containing the ancestral strain was mixed with nine volumes of acidic or neutral ASE2 or UPW, and incubated at 25 °C for 24 h. Viral RNA was isolated using ISOGEN-LS (NIPPON Genetics Co., Ltd., Tokyo, Japan), and cDNA was synthesized using FastGene cDNA Synthesis 5× ReadyMix OdT (NIPPON Genetics Co., Ltd.). Conventional PCR was performed for 30 cycles using GoTaq^®^ Green Master Mix (Promega Co.). Subsequent agarose gel electrophoresis was performed on a 1.5% agarose gel with pHY marker (Takara Bio Inc., Kusatsu, Japan) as a DNA marker. The primers used for conventional PCR are as follows: forward primer: 5′-TGGAGGAGGTCTTATCAGAGGCAC-3′; reverse primer: 5′-GTGTATGCCCCTCCGTTAAGCTCA-3′ (target region: ORF1ab, amplicon size: 445 bp). Real-time PCR was performed for 45 cycles using FastGene™ QPCR Probe Mastermix w/ROX (NIPPON Genetics Co., Ltd.). The primers and probe used for the real-time PCR were as previously described by Shirato et al. [26]: NIID_2019-nCoV_N_F2, NIID_2019-nCoV_N_R2, and NIID_2019-nCoV_N_P2 (target region: N gene, amplicon size: 157 bp).

### 2.6. Transmission Electron Microscopy

As there was a limitation to the electron microscopic analysis of SARS-CoV-2 from the perspective of pathogen safety management, bovine coronavirus (BCoV), which belongs to the genus *Betacoronavirus* like SARS-CoV-2, was used as a surrogate virus for the experiment. BCoV and SARS-CoV-2 share similar structures, and their S protein epitopes show a degree of homology [27]. Purified BCoV was incubated with nine volumes of either neutral ASE2 or UPW for 6 h; then, the samples were prepared for electron microscopy on 400-mesh carbon-coated collodion grids (NISSHIN EM Co., Ltd., Tokyo, Japan), in accordance with a two-step protocol [28]. The virus was negatively stained for 2 min with 2% phosphotungstic acid (pH 6.5), and the grids were examined using an H7500 transmission electron microscope (Hitachi High-Technologies Co., Tokyo, Japan). To evaluate the number of intact virions in the UPW- and neutral ASE2-treated groups, 0.52 µm^2^ fields (20 in number) were randomly chosen for three samples of each group, and the total number of intact virions was counted per 20 fields for each sample. Virions with discernible S proteins, a well-defined, undisrupted envelope, and a uniformly clear color showing no staining agent penetration were judged as intact virions.

### 2.7. Statistical Analysis

To evaluate the statistical significance of the virucidal activity of multiple test solutions, a one-way analysis of variance (ANOVA) followed by Tukey’s multiple comparison test or Kruskal–Wallis test with Dunn’s multiple comparison test was performed among three or four groups. To evaluate the statistical significance of the real-time RT-PCR findings, a one-way ANOVA followed by Tukey´s multiple comparison test was performed among the UPW, acidic ASE2, and neutral ASE2 groups. Additionally, to identify statistically significant differences between the number of intact virions observed by electron microscopy for the UPW and neutral ASE2 groups, Student’s *t* test was performed between the two groups; *p* value < 0.05 was considered statistically significant.

## 3. Results

### 3.1. Composition of ASE1

**Figure 2:**
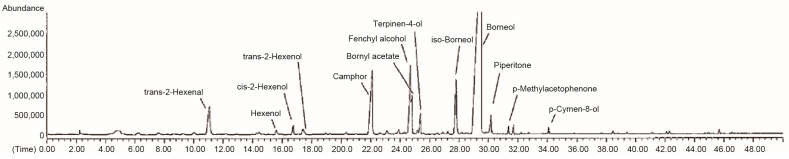
Gas chromatography–mass spectrum of ASE1.

Gas chromatography–mass spectrometry analysis revealed a composition of 84.89% monoterpenoids, 4.67% aldehydes, 2.79% alcohols, 0.04% phenolic compounds, and 7.5% other substances. Borneol, a bicyclic monoterpene, accounted for 55% of the total ASE1 composition (Table 1, Figure 2).

### 3.2. Composition of ASE2

**Figure 3:**
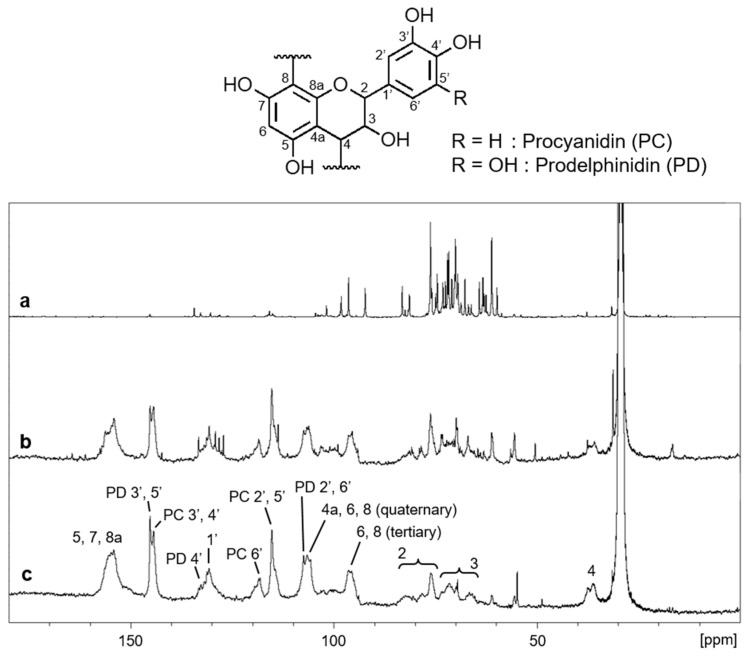
^13^C−NMR spectra of H_2_O eluates (**a**), 50% MeOH eluates (**b**), and 70% acetone eluates (**c**) separated from ASE2.

ASE2 contained nonvolatile compounds at 8.4 mg/mL. The yield and polyphenol content of the fractions separated from the nonvolatile compounds are shown in Table 2. H_2_O eluates were the most abundant fraction and mainly contained fructose and glucose with respect to authentic sugars (Figure S1). The 50% MeOH and 70% acetone eluates were polyphenol-rich fractions. ^13^C–NMR analyses revealed that 50% MeOH and 70% acetone eluates mainly contained procyanidin- and prodelphinidin-type condensed tannins [29,30] (Figure 3).

### 3.3. Virucidal Activity of ASE1 and ASE2 against SARS-CoV-2

**Figure 4:**
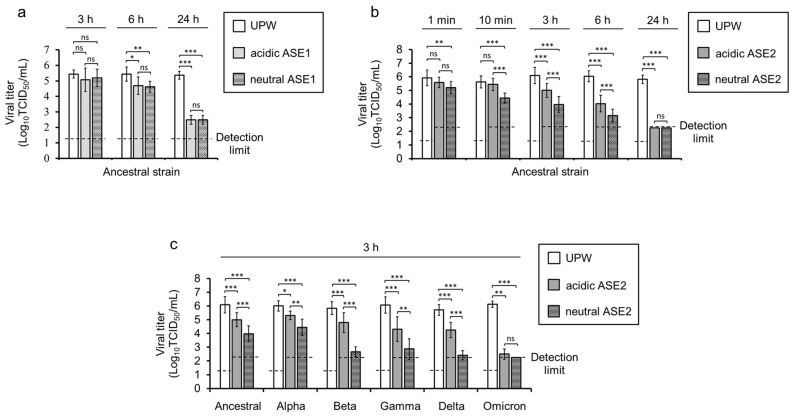
Virucidal activity of ASE1 and ASE2 against SARS-CoV-2. (**a**–**c**) SARS-CoV-2 ancestral strain (**a**,**b**) and variant strains (**c**) were mixed with nine volumes of UPW, acidic, or neutral ASE1 (**a**) or nine volumes of UPW, acidic, or neutral ASE2 (**b**,**c**). The mixtures were incubated at 25 °C from 1 min to 24 h (**a**,**b**) or for 3 h (**c**); then, the viral titers were evaluated. The data are expressed as mean ± SD (n = 8–16 per group). One-way ANOVA followed by Tukey´s multiple comparison test or Kruskal–Wallis test with Dunn’s multiple comparison test was performed to evaluate the statistical significance among the three groups. * *p* < 0.05; ** *p* < 0.01; *** *p* < 0.001; ns: not significant.

The virucidal activity of ASE1 and ASE2 against the ancestral strain was evaluated. Both acidic and neutral ASE1 and ASE2 showed a time-dependent virucidal activity against SARS-CoV-2. Before 6 h incubation, no statistically significant reduction in viral titer was observed in the acidic and neutral ASE1 groups. However, after 6 h, 0.75 and 0.81 log_10_ TCID_50_/mL viral titer reductions were observed in the acidic and neutral ASE1 groups, respectively. At 24 h incubation, both acidic and neutral ASE1 treatments led to a 2.8 log_10_ TCID_50_/mL viral titer reduction but did not reduce the viral titer to the detection limit (Figure 4a). On the other hand, ASE2 showed a stronger virucidal activity than ASE1, with a statistically significant 0.7 log_10_ TCID_50_/mL viral titer reduction in the neutral ASE2 group after 1 min incubation. Neutral ASE2 showed a significantly stronger virucidal activity than acidic ASE2 at 10 min–6 h of incubation. At these incubation times, the viral titer reduction in the neutral ASE2 group was always superior to that in the acidic ASE2 group by about 1.0 log_10_ TCID_50_/mL. At 24 h incubation, both acidic and neutral ASE2 reduced the viral titer to under the detection limit, with a ≥3.5 log_10_ TCID_50_/mL viral titer reduction (Figure 4b). Neutral ASE2 also showed a stronger virucidal activity than acidic ASE2 on multiple variant strains. At 3 h incubation, neutral ASE2 reduced the viral titer of variant strains close to the detection limit, except for the Alpha variant, where the viral titer reduction was similar to that of the ancestral strain. When incubated with neutral ASE2, Beta, Gamma, and Delta variants showed a viral titer reduction (around 3.0 log_10_ TCID_50_/mL reduction) two times higher than that of the Alpha variant (1.5 log_10_ TCID_50_/mL reduction). Out of all the variants, the Omicron variant showed the highest viral titer reduction by both acidic and neutral ASE2 treatments, and it was the only variant where both acidic and neutral ASE2 reduced the viral titer to close to the detection limit, with a ≥3.6 log_10_ TCID_50_/mL and ≥3.8 log_10_ TCID_50_/mL viral titer reduction, respectively (Figure 4c).

### 3.4. Impact of pH on the Virucidal Activity of ASE2

**Figure 5:**
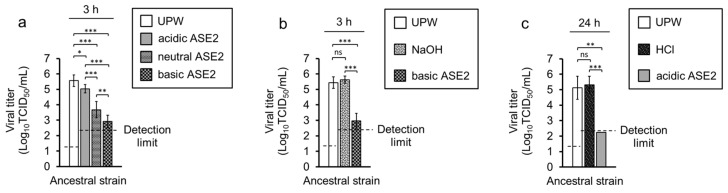
Impact of pH on the virucidal activity of ASE2 against SARS-CoV-2. (**a**–**c**) SARS-CoV-2 ancestral strain was mixed with nine volumes of UPW, acidic, neutral, or basic ASE2 (**a**), nine volumes of UPW, NaOH solution (pH 10.0), or basic ASE2 (**b**), or nine volumes of UPW, HCl solution (pH 2.9), or acidic ASE2 (**c**). The estimated final pH of the mixtures containing NaOH or basic ASE2 was 8.7; it was 5.1 in the mixtures containing HCl or acidic ASE2. After 3 h (**a**,**b**) or 24 h (**c**) incubation at 25 °C, the viral titer in each mixture was evaluated. The data are expressed as mean ± SD (n = 8 per group). One-way ANOVA followed by Tukey´s multiple comparison test or Kruskal–Wallis test with Dunn’s multiple comparison test was performed to evaluate the statistical significance among four (**a**) or three (**b**,**c**) groups. * *p* < 0.05; ** *p* < 0.01; *** *p* < 0.001; ns: not significant.

A positive correlation was observed between increased pH and the virucidal activity of ASE2. When the test solutions were incubated with the ancestral strain for 3 h, basic ASE2 showed the strongest virucidal activity, followed by neutral ASE2 and acidic ASE2 (Figure 5a). However, the observed virucidal activity did not appear to be solely caused by pH, as neither the NaOH nor HCl controls showed any virucidal activity against the SARS-CoV-2 ancestral strain at 3 h and 24 h, respectively (Figure 5b,c).

### 3.5. Impact of ASE2 on SARS-CoV-2 Structural Proteins

**Figure 6:**
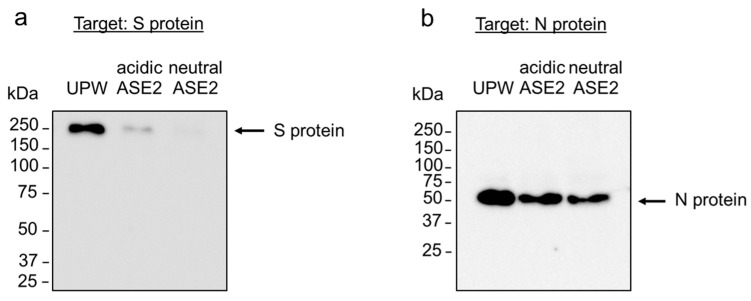
Impact of ASE2 on SARS-CoV-2 structural proteins. (**a**,**b**) SARS-CoV-2 ancestral strain was mixed with nine volumes of UPW, acidic, or neutral ASE2. After 24 h incubation at 25 °C, WB targeting the S (**a**) and N (**b**) proteins of SARS-CoV-2 was performed. The experiment was repeated six times for the S protein and three times for the N protein.

The impact of ASE2 on SARS-CoV-2 S and N proteins on the ancestral strain was investigated using WB. Probing the PVDF membrane with anti-S protein polyclonal antibodies revealed a single 250 kDa band identified as a multimer of S protein after 24 h incubation of UPW-treated virus. The intensity of the S protein band was weaker in the acidic ASE2-treated virus than in the UPW-treated one, and it completely disappeared in the neutral ASE2-treated one (Figure 6a). Probing the PVDF membrane with anti-N protein monoclonal antibodies revealed a single 50 kDa band after 24 h incubation of UPW-treated virus. Both acidic ASE2- and neutral ASE2-treated viruses showed a thinner band than the UPW-treated one (Figure 6b).

### 3.6. Impact of ASE2 on SARS-CoV-2 Genome

**Figure 7:**
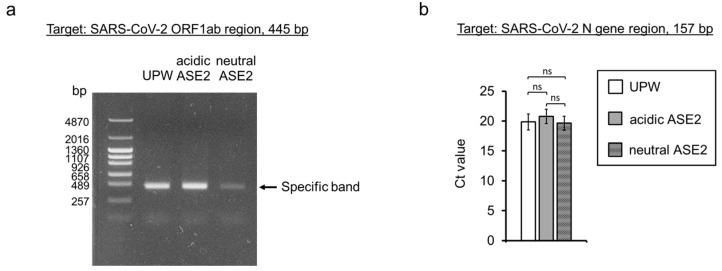
Impact of ASE2 on the SARS-CoV-2 genome. (**a**,**b**) SARS-CoV-2 ancestral strain was mixed with nine volumes of UPW, acidic, or neutral ASE2. After 24 h incubation at 25 °C, conventional RT-PCR (**a**) and real-time RT-PCR (**b**) were performed. (**a**) The conventional PCR primer set amplified a 445 bp fragment in the ORF1ab region. Conventional RT-PCR was repeated five times. (**b**) The real-time PCR primer set amplified a 157 bp fragment in the N gene region. The Ct values are expressed as mean ± SD (n = 4 per group). One-way ANOVA followed by Tukey’s multiple comparison test was performed to evaluate the statistical significance among the three groups. ns: not significant.

The impact of ASE2 on the SARS-CoV-2 genome was evaluated using conventional and real-time RT-PCR after incubating the ancestral strain with UPW, acidic, or neutral ASE2 for 24 h. After conventional RT-PCR, a reduction in PCR product band intensity was observed for the neutral ASE2-treated virus compared to the ones treated with UPW and acidic ASE2 (Figure 7a). Conversely, no significant difference was observed between the cycle threshold (Ct) value of the UPW-treated group compared with that of the acidic and neutral ASE2-treated groups in real-time RT-PCR (Figure 7b).

### 3.7. Impact of ASE2 on Betacoronavirus Virions

**Figure 8:**
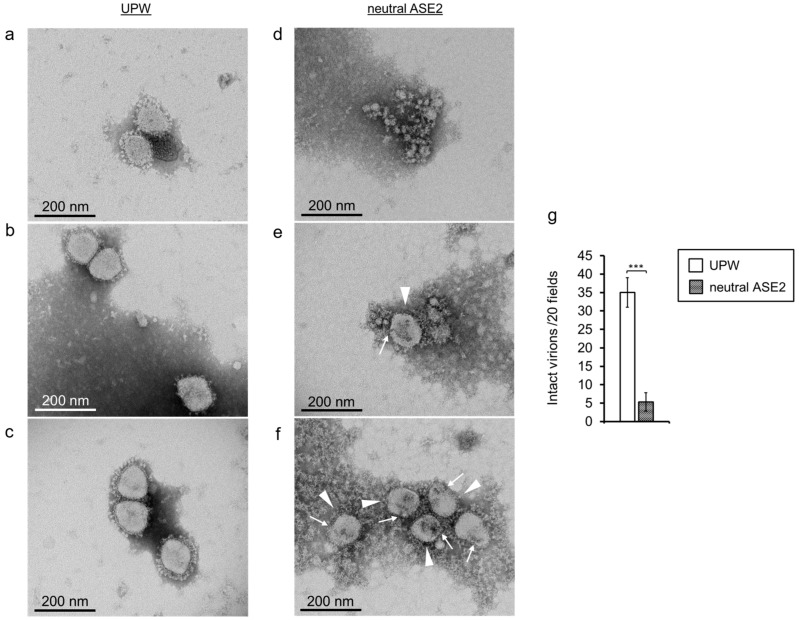
Impact of neutral ASE2 on Betacoronavirus virions. (**a**–**g**) BCoV, a surrogate of SARS-CoV-2, was incubated with nine volumes of UPW or neutral ASE2. After 6 h incubation at 25 °C, the virions were observed using electron microscopy. (**a**–**c**) BCoV virions incubated with UPW; (**d**–**f**) BCoV virions incubated with neutral ASE2. The arrowheads indicate virions on which S protein structure could not be clearly observed. The arrows show areas of the envelope where envelope integrity was disrupted and the staining agent penetrated the virions. (**g**) The number of intact virions was counted within 0.52 µm^2^ fields (20 in number) chosen randomly in three samples of each group. The results are indicated as mean ± SD (n = 3 per group). Virions with discernible S proteins, well-defined undisrupted envelope, and a uniformly clear color showing no staining agent penetration were judged as intact virions. Student’s *t* test was performed to evaluate the statistical significance of differences between the two groups; *** *p* < 0.001.

BCoV was used as a surrogate to SARS-CoV-2. BCoV virions were observed using a transmission electron microscope after 6 h incubation with UPW or neutral ASE2. In the UPW group, virions maintained a normal virion structure with clearly discernible S protein and a well-defined, intact envelope (Figure 8a–c). In the virions of the neutral ASE2-treated group, S protein structure was unclear in the majority of virions. Envelope integrity appeared to be disrupted in some areas, with the staining agent having penetrated the virions, which could be observed by the presence of dark spots inside them (Figure 8d–f). Additionally, the number of intact virions observed in the neutral ASE2-treated group was significantly lower than that in the UPW group (Figure 8g).

## 4. Discussion

In this study, the virucidal activity of two aqueous extracts from *A. sachalinensis* branches and leaves, ASE1 and ASE2, against SARS-CoV-2 was investigated. ASE1 did not significantly reduce the viral titer before 6 h incubation, and did not reduce the viral titer to the detection limit in 24 h (Figure 4a). In contrast, ASE2 induced a statistically significant viral titer reduction after 1 min incubation, and reduced the viral titer to under the detection limit in 24 h (Figure 4b). Furthermore, a positive correlation between the increase in pH and virucidal activity was observed for ASE2 but not for ASE1 (Figure 4a,b and Figure 5a). However, HCl and NaOH control solutions, which were expected to show a similar final pH to acidic and basic ASE2, respectively, after mixing with the virus solution, did not exhibit any viral titer reductions (Figure 5b,c). Thus, the stronger virucidal activity of ASE2 in increased pH conditions was linked to the compounds inside this extract rather than to pH alone.

To our knowledge, this study is the first to report an increased virucidal activity of an acidic plant extract against SARS-CoV-2 upon an increase in its natural pH. However, sparse reports of a positive correlation between antimicrobial activity of plant extracts and increased pH can be found in the literature. In a study by Shahi et al. [31], four essential oils from *Eucalyptus* spp. exhibited a higher antifungal activity when their original acidic pH of 5.6 was increased to neutral or basic. Their major constituent was cineole, also called eucalyptol, a bicyclic monoterpene [31,32]. In our study, the major constituent of ASE1 was borneol, another bicyclic monoterpene (Table 1, Figure 2). However, no difference in virucidal activity was observed between the original acidic ASE1 and neutral ASE1. Monoterpenes are vulnerable to acid-catalyzed reactions, particularly at ambient or high temperatures [33]. Possible transformations of terpenes include isomerization into compounds of different biological activities, cyclization, hydration, and oxidation [34], all of which, except oxidation, can occur in monoterpenes as a direct result of exposure to acidic aqueous conditions [33]. However, while possible transformations of monoterpenes have been widely studied in acidic aqueous conditions, there are no data about their stability in neutral or basic aqueous solutions and how it might compare to acidic solutions. In our study, the absence of a difference between the virucidal activity of acidic and neutral ASE1 suggests that even if ASE1 monoterpenoid compounds had undergone unknown transformations at increased pH, these transformations did not impact their virucidal activity against SARS-CoV-2.

Another report of a positive correlation between increased pH and antimicrobial activity was found in a study on cinnamon oil by Chang et al. [35], where potent antibacterial activity against *Legionella pneumophila* was observed when the pH of cinnamon oil was increased to basic. The major constituent of cinnamon oil is cinnamaldehyde [35], a phenolic compound and the reduced form of cinnamic acid, a flavonoid precursor [36,37]. Flavonoids are a family of plant-derived polyphenolic compounds [38]. In *Abies* sp. conifers, flavonoids are major constituents, along with terpenoids and lignans [5]. Most flavonoids are present in plants in the form of glycosides [39], which are nonvolatile [40]. In our study, ASE2 contained nonvolatile flavonoids, mainly procyanidin- and prodelphinidin-type condensed tannins (Table 2, Figure 3). We previously reported that condensed tannins showed rapid and potent virucidal activity against multiple viruses including SARS-CoV-2 [41], which suggests that the condensed tannins in ASE2 likely played an important role in SARS-CoV-2 inactivation. The stability of polyphenolic compounds has been extensively studied under different pH conditions. Many polyphenols, including condensed tannin oligomers, are unstable at an increased pH [42,43,44]. Mild alkaline pH was reported to trigger the rearrangement of condensed tannins, and cleavage of their interflavonoid bonds into flavan-3-ol monomers, which, under a neutral or alkaline pH, undergo epimerization and rearrangement into other polyphenolic compounds with quinone intermediate products [45]. It can be hypothesized that, in our experiment, increasing the pH of ASE2 from acidic to neutral could have led to the aforementioned polyphenol transformations, with implications for the virucidal activity of ASE2 at different pH.

Both polyphenols and quinones are potent redox compounds that readily interact with proteins [38,46,47,48]. Protein binding with polyphenols and quinones can result in the modification of protein structure and formation of cross-linked protein aggregates, for example, of membrane proteins [46,49]. In our study, most Betacoronavirus virions treated with neutral ASE2 had an unclear S protein structure under electron microscopic observation (Figure 8d–f). Furthermore, WB showed a reduction in the band intensity of S and N proteins in ASE2-treated viruses (Figure 6). This phenomenon was most remarkable in the group treated with neutral ASE2 rather than acidic ASE2, and the surface S protein was also more affected than the inner N protein. Because these results were observed to be more significant in neutral ASE2-treated samples, transformations of ASE2 condensed tannins under an increased pH could have resulted in compounds with a better affinity for SARS-CoV-2 proteins in neutral ASE2.

Additionally, envelope disruption was also observed for Betacoronavirus virions treated with neutral ASE2 (Figure 8d–f). The viral envelope is a lipid bilayer derived from cell membranes, and both polyphenolic compounds and quinones have been reported to interact with lipid bilayers [50,51]. Flavonoids such as flavan-3-ols, the components of condensed tannins, trigger phospholipid aggregation within lipid membranes and subsequent membrane rigidity, resulting in the bursting of lipid vesicles [52]. Quinones have also been reported to penetrate lipid bilayers and perturb their biophysical properties [50] and disrupt the viral envelope [53]. Therefore, the interaction between ASE2 condensed tannins and quinone transformation products with the SARS-CoV-2 envelope could explain the envelope disruption observed under electron microscopy.

Neutral ASE2 also affected the amplification of a 445 bp viral genome fragment in conventional RT-PCR, while acidic ASE2 did not (Figure 7a). While polyphenols are generally considered beneficial to human health, both polyphenols and quinones have been reported to exert DNA damage through pro-oxidant activity, the extent of which appears to be linked to compound structure and dosage [48,54,55]. pH-induced differences in the polyphenolic compound composition of neutral ASE2 compared with that of acidic ASE2 could, therefore, explain the observed difference. However, upon amplifying a shorter genome fragment of 157 bp through real-time RT-PCR, no significant difference was observed between the ASE2-treated groups and the UPW group (Figure 7b), which suggests that genome damage by ASE2 is not extensive and only detected upon amplifying longer genome fragments.

In our experiments, among the multiple tested strains, the Omicron variant was the most susceptible to ASE2 (Figure 4c). An in silico study on SARS-CoV-2 and curcumin by Nag et al. [56] reported a strong binding affinity of curcumin, a polyphenol, to the S protein of the Omicron variant. Nine substitutions in the amino acid sequence of the Omicron S protein were determined to be the cause of that high binding affinity [56], among which only two, LYS478 and TYR501, are not exclusive to the Omicron variant [57]. Additionally, in another molecular docking study by Schultz et al. [58] on the binding affinity of two flavonoids, apigenin and orientin, to the S protein of the Omicron and Delta variants, both flavonoids exhibited a stronger binding affinity to the Omicron S protein than to the Delta S protein. Moreover, a two-dimensional map of interactions between the flavonoids and S proteins revealed more hydrogen bonding sites for the Omicron S protein than there were for the Delta S protein [58]. Thus, the Omicron S protein appears to be more susceptible to the action of polyphenols than the S protein of older variants, which could explain the increased virucidal activity of ASE2 toward the Omicron variant.

Hence, it is hypothesized that nonvolatile polyphenols caused the observed virucidal activity of ASE2 against SARS-CoV-2, and that a pH increase induced structural changes in these polyphenols that impacted the virucidal activity of ASE2. Further experiments are needed to analyze the nature of the pH-induced structural changes in ASE2 polyphenolic compounds.

## 5. Conclusions

ASE2, the extract containing nonvolatile polyphenols, showed a stronger virucidal activity against SARS-CoV-2 than ASE1, the extract rich in volatile monoterpenoids. Additionally, modifying the original acidic pH of ASE2 increased its effectiveness as a virucidal agent; a positive correlation between pH increase and virucidal activity was observed. A neutral ASE2 led to a significant viral titer reduction in SARS-CoV-2 ancestral and variant strains, affected the viral S and N proteins, and disrupted the SARS-CoV-2 genome. Neutral ASE2 also induced envelope disruption in Betacoronavirus virions. Thus, modifying the pH of ASE2 led to the discovery of its full potential as a SARS-CoV-2 virucidal agent. Future applications of ASE2 to create naturally derived disinfectant products against SARS-CoV-2 can be envisioned to control virus spread and infections, such as using neutral or basic ASE2, or extracted ASE2 active compounds, for a wider range of applications. The utilization of ASE2 or its active compounds could bring much-needed value to *A. sachalinensis* wood byproducts.

## Figures and Tables

**Table 1:** Composition of ASE1.

Compound Name	Area (%)	CAS No.
Borneol	55.035	464-45-9
Camphor	8.785	76-22-2
Bornyl acetate	5.903	5655-61-8
iso-Borneol	5.903	124-76-5
trans-2-Hexenal	4.243	6728-26-3
Fenchyl alcohol	2.628	1632-73-1
Piperitone	1.375	89-81-6
Terpinen-4-ol	1.340	562-74-3
cis-3-Hexenol	0.756	928-96-1
Fenchone	0.549	1195-79-5
Hexanol	0.503	111-27-3
p-Methylacetophenone	0.425	122-00-9
Pinocamphone	0.389	547-60-4
Pinocarvone	0.384	30460-92-5
1,8-Cineole	0.360	470-82-6
p-Cymen-8-ol	0.329	1197-01-9
1,4-Cineole	0.329	470-67-7
Camphene hydrate	0.322	465-31-6
Cryptone	0.296	500-02-7
Hexanal	0.246	66-25-1
2-Heptanol	0.244	543-49-7
Linalool oxide (Furanoid, p-2)	0.217	60047-17-8
Carvacrol	0.213	499-75-2
Pinocarveol	0.210	547-61-5
trans-2-Pentenal	0.187	1576-87-0
1-Penten-3-ol	0.176	616-25-1
4-iso-Propylcyclohexanol	0.159	4621-04-9
trans-2-Hexenol	0.151	928-95-0
Carvone	0.145	6485-40-1
Cumin alcohol	0.145	536-60-7
Benzaldehyde	0.135	100-52-7
cis-2-Pentenol	0.121	1576-95-0
Filifolone (p-2)	0.116	4613-37-0
Pinol	0.113	2437-97-0
Myrtenol	0.104	19894-97-4
Linalool oxide acetate	0.097	67674-42-4
3-Pentanone	0.092	96-22-0
Verbanone	0.087	1196-01-6
Linalool oxide (Furanoid, p-1)	0.086	60047-17-8
trans-3-Hexenol	0.084	928-97-2
Maltol	0.067	118-71-8
Camphene	0.065	79-92-5
2,6,6-Trimethyl-2-vinyltetrahydropyran	0.064	7392-19-0
Thymol	0.045	89-83-8
n-Amyl alcohol	0.044	71-41-0
Perillaldehyde	0.038	18031-40-8
Carveol (p-1)	0.037	99-48-9
Dehydro-p-cymene	0.036	1195-32-0
Filifolone (p-1)	0.034	4613-37-0
Limonene	0.033	5989-27-5
α-Terpineol	0.033	98-55-5
p-iso-Propylphenol	0.026	99-89-8
Piperitone	0.019	89-81-6
Benzyl alcohol	0.018	100-51-6
Methyleugenol	0.018	93-15-2
p-Cresol	0.016	106-44-5
Carveol (p-2)	0.011	99-48-9
Anisaldehdye	0.010	123-11-5
Acetic acid	0.008	64-19-7
p-Cymene	0.003	99-87-6
unidentified peaks	6.396	

**Table 2:** Yield and polyphenol content of the fractions separated from the nonvolatile compounds in ASE2.

			Fraction yield (%)		Polyphenol content (%)
			/ASE2	/NVC	/Each Fr.
H_2_O insolubles			0.06	6.7	0.2
H_2_O solubles			0.78	93.3	3.4
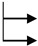	70% acetone insolubles		0.19	24.2	2.6
	70% acetone solubles		0.50	64.5	4.1
	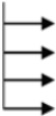	EtOAc solubles	0.12	15.2	5.3
		H_2_O eluates	0.37	47.2	4.6
		50% MeOH eluates	0.01	0.8	22.4
		70% acetone eluates	0.0002	0.03	10.2

Yield and content are expressed as weight percentage based on ASE2 (/ASE2), nonvolatile compounds (/NVC) and each fraction (/Each Fr.).

## Data Availability

The original contributions presented in the study are included in the article. Further inquiries can be directed to the corresponding author.

## Supplementary Materials

The following supporting information can be downloaded at: https://www.mdpi.com/article/10.3390/pathogens12091093/s1, Figure S1: ^13^C−NMR spectra of sucrose (a), fructose (b), glucose (c), and H_2_O eluates from ASE2 (d).
