# Supplementary material for: An Abies Extract Containing Nonvolatile Polyphenols Shows Virucidal Activity against SARS-CoV-2 That Is Enhanced in Increased pH Conditions
Source: Pathogens. 2023 Aug 28;12(9):1093. doi: 10.3390/pathogens12091093 (PMC10534523; doi:10.3390/pathogens12091093)
Supplement: Supplementary file 1 [file pathogens-12-01093-s001.zip › pathogens-2471608-supplementary.pdf]

Supplementary Materials for **An *Abies* extract containing nonvolatile polyphenols shows virucidal activity against SARS-CoV-2 that is enhanced in increased pH conditions**

Imane Maaroufi [1], Dulamjav Jamsransuren [2], Koh Hashida [3], Sachiko Matsuda [2], Haruko Ogawa [4] and Yohei Takeda [2,4,*]

[1] Graduate School of Animal and Veterinary Sciences and Agriculture, Obihiro University of Agriculture and Veterinary Medicine, Obihiro 080-8555, Japan; imanemaaroufivet@gmail.com
[2] Research Center for Global Agromedicine, Obihiro University of Agriculture and Veterinary Medicine, Obihiro 080-8555, Japan; jduuya@obihiro.ac.jp (D.J.); chaka@obihiro.ac.jp (S.M.); ytakeda@obihiro.ac.jp (Y.T.)
[3] Department of Forest Resources Chemistry, Forestry and Forest Products Research Institute, Tsukuba 305-8687, Japan; koh@ffpri.affrc.go.jp (K.H.)
[4] Department of Veterinary Medicine, Obihiro University of Agriculture and Veterinary Medicine, Obihiro 080-8555, Japan; hogawa@obihiro.ac.jp (H.O.)
* Correspondence: ytakeda@obihiro.ac.jp; Tel.: +81-155-49-5896

## Supplementary Figure

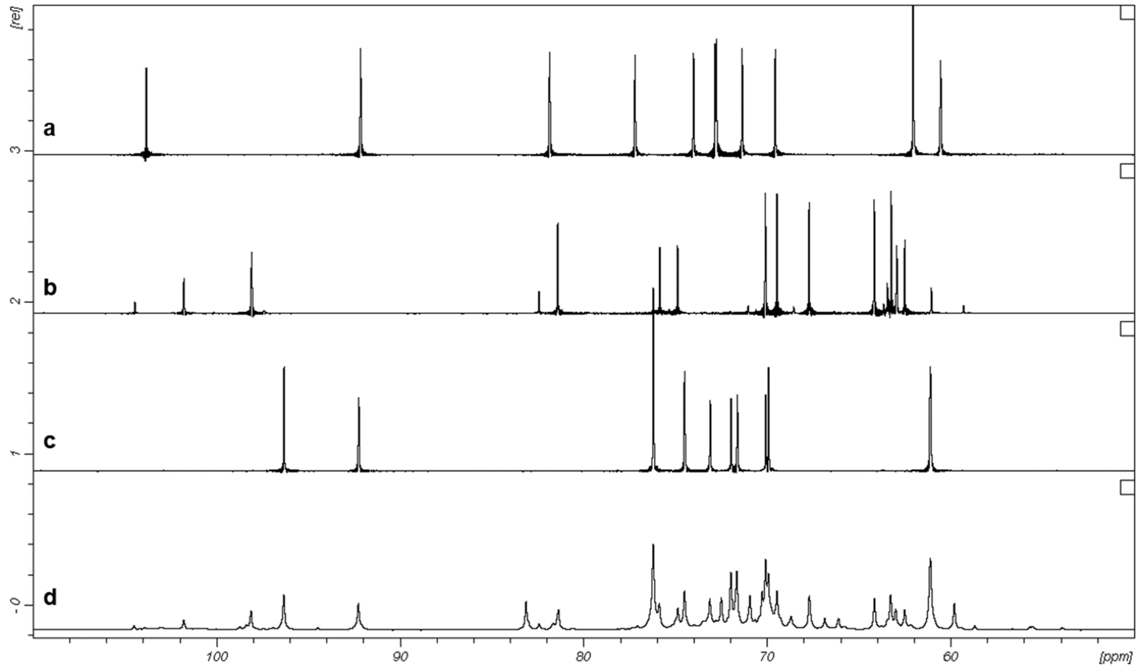


**Figure S1.** $^{13}$C−NMR spectra of sucrose (**a**), fructose (**b**), glucose (**c**), and $H_2O$ eluates from ASE2 (**d**).
